# Supplementary material for: Drug repurposing for aging research using model organisms
Source: Aging Cell. 2017 Jun 16;16(5):1006–15. doi: 10.1111/acel.12626 (PMC5595691; doi:10.1111/acel.12626)
Supplement: Supplementary file 7 — Data S1 Zip‐Archive of all report cards. [file ACEL-16-1006-s007.zip › RC_MES.pdf]

MES

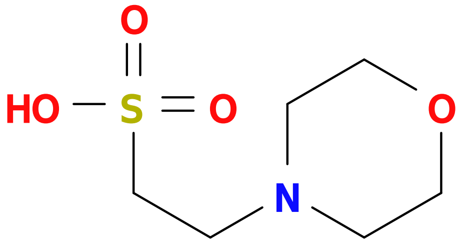

Database identifiers

ChEMBLCompound	CHEMBL1234276
DrugBank	DB03814
CHEBI	39005
CHEBI	39408
ZINC	ZINC01583444
eMolecules	529295

Ranking

	Rank	Score
Drosophila	324/697	0.482
C. elegans	271/591	0.205

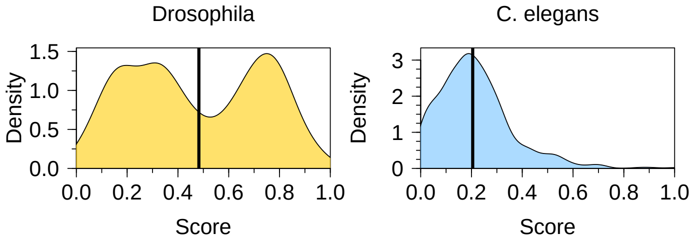

	Ageing implication	Domain conservation	Binding site conservation	Binding affinity	Bioavailability	Lipinski	Promiscuity	Purchasability	Drug approval	**Total**
Drosophila	1.0	0.978	1.0	0.41	(0.9)	0.0	-0.054	0.1	0.075	0.482
C. elegans	1.0	0.951	1.0	0.41	0.214	0.0	-0.054	0.1	0.075	0.205

Names

- **2-(N-morpholiniumyl)ethanesulfonate**
- 2-(morpholin-4-ium-4-yl)ethanesulfonate
- 2-(N-Morpholino)-Ethanesulfonic Acid

Roles

ChEBI entry 39408 has no roles

Status

Approved drug (according to ChEMBL)	No
Classification (according to DrugBank)	experimental
Number of Rule of 5 violations	0
Binding affinity to original target in log units (RF-Score prediction)	4.64
Burns *C. elegans* bioavailability prediction	-5.0

Compound Target Characteristics

Cyclin-dependent protein kinase PHO85

Best gene implication in ageing for this target family came from gene P48609 annotated in UniProt release 2014_02. Annotation GO 8340 (determination of adult lifespan) was Inferred from Mutant Phenotype

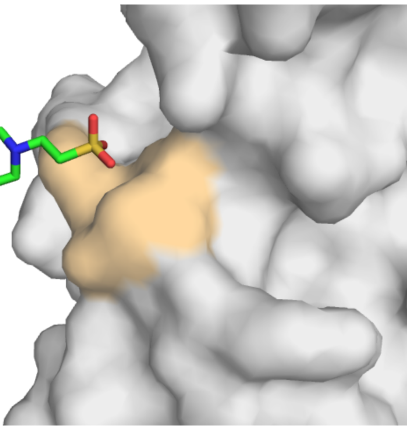

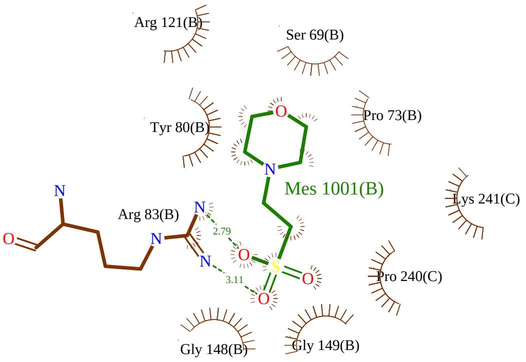

protein	amino acids contacts (binding site)		
PDB:2pk9:chainA:P17157	G	P	K
PDB:2pk9:chainC:P17157	G	P	K
PDB:2pmi:chainA:P17157	G	P	K
sp:Q00535:CDK5_HUMAN	G	P	D
sp:Q03114:CDK5_RAT	G	P	D
sp:P49615:CDK5_MOUSE	G	P	D
tr:Q543F6:Q543F6_MOUSE	G	P	D
sp:P48609:CDK5_DROME	G	S	D
tr:G5ECH7:G5ECH7_CAEEL	G	P	D
sp:P17157:PHO85_YEAST	G	P	K

	whole protein		domain-based		contact-based	
protein	ident	simil	ident	simil	ident	simil
PDB:2pk9:chainA:P17157	1.0	1.0	1.0	1.0	1.0	1.0
PDB:2pk9:chainC:P17157	1.0	1.0	1.0	1.0	1.0	1.0
PDB:2pmi:chainA:P17157	1.0	1.0	1.0	1.0	1.0	1.0
sp:Q00535:CDK5_HUMAN	0.54	0.82	0.56	0.83	0.67	0.53
sp:Q03114:CDK5_RAT	0.53	0.81	0.55	0.83	0.67	0.53
sp:P49615:CDK5_MOUSE	0.54	0.81	0.56	0.83	0.67	0.53
tr:Q543F6:Q543F6_MOUSE	0.54	0.81	0.56	0.83	0.67	0.53
sp:P48609:CDK5_DROME	0.51	0.81	0.53	0.81	0.33	0.53
tr:G5ECH7:G5ECH7_CAEEL	0.51	0.8	0.52	0.81	0.67	0.53
sp:P17157:PHO85_YEAST	1.0	1.0	1.0	1.0	1.0	1.0

Cdk5 (FBgn0013762) associated phenotypes

neuroanatomy defective, short lived

(Information from FlyBase)

Cdk5 (UniProt:P48609) annotation

Function: Probably involved in the control of the cell cycle. Interacts with D1 and D3-type G1 cyclins. Possible regulator of neuronal differentiation and/or development (By similarity).

Tissue specificity: Abundantly expressed in all adult tissues. Lower levels found in larvae and early embryos. Barely detectable in late embryos.

(Information from UniProt)

cdk-5 (WBGene00000407) associated phenotypes
aldicarb resistant

(Information from WormBase)

cdk-5 (UniProt:G5ECH7) annotation
Function: Proline-directed serine/threonine-protein kinase which, in several motor neurons, promotes the polarized trafficking of synaptic vesicles and dense-core vesicles (DCV). In the ventral nerve cord, phosphorylates lin-10 and thereby prevents lin-10- mediated anterograde trafficking of the glutamate receptor glr-1 (PubMed:17671168, PubMed:21609829). Involved in the inhibition of glr-1 trafficking in hypoxic conditions (PubMed:22252129). In DA motor neurons but not in DB motor neurons, regulates axonal transport of synaptic vesicle precursors by inhibiting dynein-mediated retrograde transport (PubMed:20510931). Regulates the trafficking of dense-core vesicles in DA and DB motor neurons by promoting anterograde trafficking to the axon and preventing dynein-dependent trafficking to the dendrite (PubMed:22699897). May regulate these processes in association with cdka-1/p35 (PubMed:17671168, PubMed:20510931). Activity may be regulated by cyy-1 (PubMed:20510931). Involved in synapse formation during DD motor neuron remodeling by regulating transport of disassembled synaptic material to the new synaptic sites probably by activating the motor protein unc-104/kinesin-3 (PubMed:21609829). Regulates microtubule polarity in the dendrite of DB motor neurons (PubMed:22699897). (PubMed:17671168, PubMed:20510931, PubMed:21609829, PubMed:22252129, PubMed:22699897).
Cofactor: Mg(2+) Evidence=(PubMed:20510931); Note=Binds 2 Mg(2+) ions. (UniProtKB:P24941);
Subunit: Heterodimer composed of a catalytic subunit cdk-5 and a regulatory subunit cdka-1. Interaction with cdka-1 is required for cdk-5 activation. (PubMed:22699897).
Subcellular location: Cytoplasm (PubMed:20510931). Cell projection, dendrite (PubMed:20510931). Note=Localizes predominantly to presynaptic sites and in dendrites as faint puncta. (PubMed:20510931).
Disruption phenotype: Several glr-1-dependent behaviors are affected including an absence of backward locomotion after nose- touching stimuli and a reduction in reverse locomotion (PubMed:17671168). In L4 mutants, incomplete elimination of ventral rab-3-positive synaptic vesicles associated with a delay in the formation of dorsal rab-3-positive synaptic vesicles in DD motor neurons. Normal formation of ventral synapses in DD motor neurons at the L1 stage (PubMed:21609829). In addition, mutants have an increase in anterograde dense-core vesicle trafficking and in the number of plus-end-out microtubules in DB motor neuron dendrites (PubMed:22699897). Reduced sensitivity to the acetylcholine esterase inhibitor aldicarb (PubMed:22699897). (PubMed:17671168, PubMed:21609829, PubMed:22699897).

(Information from UniProt)

6-phosphogluconate dehydrogenase, decarboxylating

Best gene implication in ageing for this target family came from gene P41572 via mapping the annotation from FlyBase FBgn0004654 via mapping the annotation from UniProt Q95NF4 via mapping the annotation from Ensembl FBgn0023169 via mapping the annotation from EntrezGene 43904 via mapping the annotation from GenAgeModels 1846 annotated in GenAge release 17.

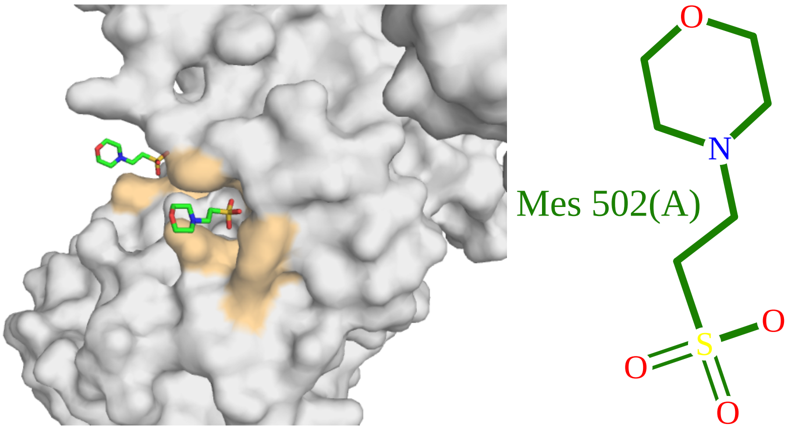

protein	amino acids contacts (binding site)
PDB:4gwg:chainA:P52209	E Y R G E M W
PDB:4gwk:chainA:P52209	E Y R G E M W
tr:B4DQJ8:B4DQJ8_HUMAN	E Y R G E M W
sp:P52209:6PGD_HUMAN	E Y R G E M W
sp:Q9DCD0:6PGD_MOUSE	E Y R G E M W
sp:P41572:6PGD_DROME	E Y Q G E M W
sp:Q17761:6PGD_CAEEL	E Y T G E M W
sp:P38720:6PGD1_YEAST	H F P G E M W
sp:P53319:6PGD2_YEAST	H F P G E M W

	whole protein		domain-based		contact-based	
protein	ident	simil	ident	simil	ident	simil
PDB:4gwg:chainA:P52209	1.0	1.0	1.0	1.0	1.0	1.0
PDB:4gwk:chainA:P52209	1.0	1.0	1.0	1.0	1.0	1.0
tr:B4DQJ8:B4DQJ8_HUMAN	0.97	0.97	0.98	0.98	1.0	1.0
sp:P52209:6PGD_HUMAN	1.0	1.0	1.0	1.0	1.0	1.0
sp:Q9DCD0:6PGD_MOUSE	0.94	0.99	0.94	0.99	1.0	1.0
sp:P41572:6PGD_DROME	0.72	0.9	0.72	0.9	0.86	0.93
sp:Q17761:6PGD_CAEEL	0.69	0.9	0.69	0.9	0.86	0.89
sp:P38720:6PGD1_YEAST	0.65	0.88	0.66	0.89	0.57	0.74
sp:P53319:6PGD2_YEAST	0.64	0.87	0.65	0.89	0.57	0.74

Pgd (FBgn0004654) associated phenotypes

maternal effect, partially lethal - majority die

(Information from FlyBase)

Pgd (UniProt:P41572) annotation

Function: Catalyzes the oxidative decarboxylation of 6- phosphogluconate to ribulose 5-phosphate and CO(2), with concomitant reduction of NADP to NADPH.
Pathway: Carbohydrate degradation; pentose phosphate pathway; D- ribulose 5-phosphate from D-glucose 6-phosphate (oxidative stage): step 3/3.
Subunit: Homodimer.

(Information from UniProt)

T25B9.9 (UniProt:Q17761) annotation

Function: Catalyzes the oxidative decarboxylation of 6- phosphogluconate to ribulose 5-phosphate and CO(2), with concomitant reduction of NADP to NADPH.
Pathway: Carbohydrate degradation; pentose phosphate pathway; D- ribulose 5-phosphate from D-glucose 6-phosphate (oxidative stage): step 3/3.
Subunit: Homodimer.

(Information from UniProt)

Serine/threonine-protein phosphatase PP1-alpha catalytic subunit

Best gene implication in ageing for this target family came from gene P62136 via mapping the annotation from Ensembl ENSG00000172531 via mapping the annotation from EntrezGene 5499 via mapping the annotation from GenAgeHuman 0227 annotated in GenAge release 17.

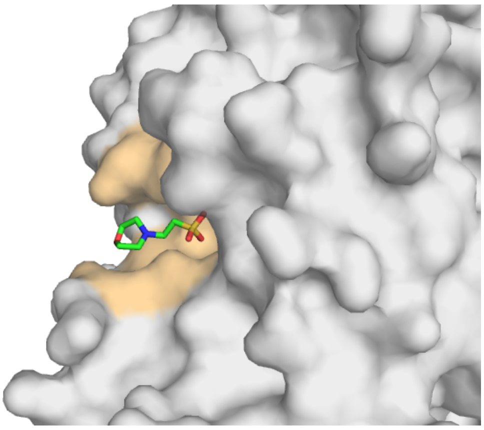

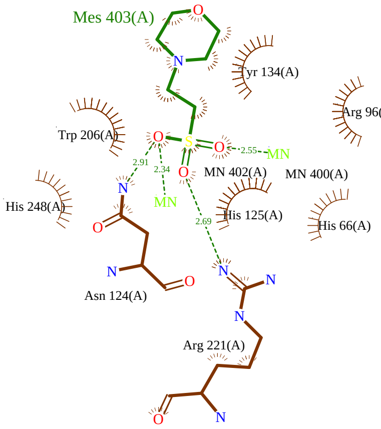

protein	amino acids contacts (binding site)
PDB:3egg:chainA:P62136	H D R N H Y W R H Y
tr:E9PMD7:E9PMD7_HUMAN	H D R N H Y W R H Y
sp:P62136:PP1A_HUMAN	H D R N H Y W R H Y
sp:P62138:PP1A_RAT	H D R N H Y W R H Y
sp:P62137:PP1A_MOUSE	H D R N H Y W R H Y
sp:P12982:PP12_DROME	H D R N H Y W R H Y
tr:B4F4X2:B4F4X2_DROME	H D R N H Y W R H Y
tr:C0MKX3:C0MKX3_DROME	H D R N H Y W R H Y
sp:Q05547:PP13_DROME	H D R N H Y W R H Y
sp:P48461:PP11_DROME	H D R N H Y W R H Y
tr:Q4W5Q9:Q4W5Q9_CAEEL	H D R N H Y W R H Y
sp:P32598:PP12_YEAST	H D R N H Y W R H Y

	whole protein		domain-based		contact-based	
protein	ident	simil	ident	simil	ident	simil
PDB:3egg:chainA:P62136	1.0	1.0	1.0	1.0	1.0	1.0
tr:E9PMD7:E9PMD7_HUMAN	0.77	0.77	0.83	0.83	1.0	1.0
sp:P62136:PP1A_HUMAN	1.0	1.0	1.0	1.0	1.0	1.0
sp:P62138:PP1A_RAT	1.0	1.0	1.0	1.0	1.0	1.0
sp:P62137:PP1A_MOUSE	1.0	1.0	1.0	1.0	1.0	1.0
sp:P12982:PP12_DROME	0.85	0.9	0.93	0.98	1.0	1.0
tr:B4F4X2:B4F4X2_DROME	0.84	0.89	0.92	0.98	1.0	1.0
tr:C0MKX3:C0MKX3_DROME	0.84	0.89	0.92	0.98	1.0	1.0
sp:Q05547:PP13_DROME	0.84	0.89	0.92	0.98	1.0	1.0
sp:P48461:PP11_DROME	0.86	0.95	0.92	0.98	1.0	1.0
tr:Q4W5Q9:Q4W5Q9_CAEEL	0.45	0.75	0.47	0.77	1.0	1.0
sp:P32598:PP12_YEAST	0.79	0.9	0.85	0.96	1.0	1.0

Pp1-87B (FBgn0004103) associated phenotypes

dominant, mitotic cell cycle defective, non-suppressor of variegation, partially lethal - majority die, somatic clone, suppressor of variegation

(Information from FlyBase)

Pp1-87B (UniProt:P12982) annotation

Function: Is essential for the regulation of mitotic chromosomal segregation as well as regulation of chromatin condensation during interphase. (PubMed:1330679, PubMed:2175717).
Cofactor: Mn(2+)Note=Binds 2 manganese ions per subunit. ;
Subunit: Interacts with Nop17l. (PubMed:17007873).

Subcellular location: Cytoplasm.

(Information from UniProt)

Pp1-13C (FBgn0003132) associated phenotypes

neuroanatomy defective

(Information from FlyBase)

Pp1-13C (UniProt:Q05547) annotation

Cofactor: Mn(2+)Note=Binds 2 manganese ions per subunit. ;
Subunit: Interacts with Nop17l. (PubMed:17007873).

(Information from UniProt)

Pp1alpha-96A (FBgn0003134) associated phenotypes

conditional, immune response defective

(Information from FlyBase)

Pp1alpha-96A (UniProt:P48461) annotation

Cofactor: Mn(2+)Note=Binds 2 manganese ions per subunit. ;
Subunit: Interacts with Nop17l. (PubMed:17007873).

(Information from UniProt)

mRNA export factor

Best gene implication in ageing for this target family came from gene A2ASC6 via mapping the annotation from Ensembl ENSMUSG00000027509 via mapping the annotation from EntrezGene 66679 via mapping the annotation from GenAgeModels 0537 annotated in GenAge release 17.

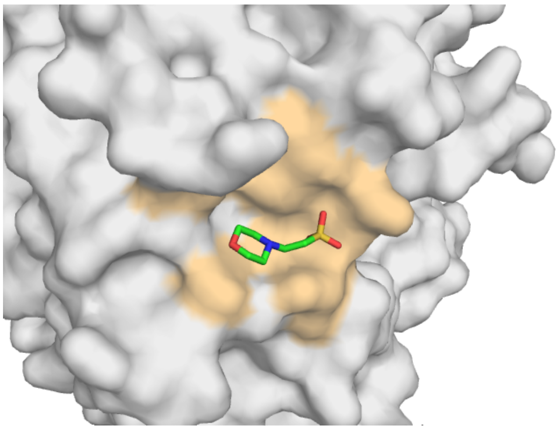

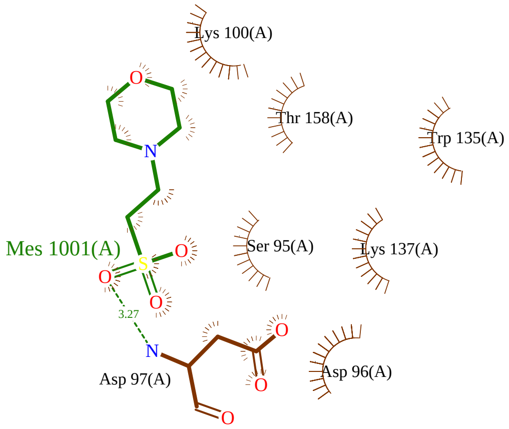

protein	amino acids contacts (binding site)
PDB:3mmy:chainA:P78406	S D D K W K S T
tr:E9PPG9:E9PPG9_HUMAN	S D D K W K S T
sp:P78406:RAE1L_HUMAN	S D D K W K S T
tr:E9PQ57:E9PQ57_HUMAN	S D D K W K S T
sp:Q3SWS8:RAE1L_RAT	S D D K W K S T
tr:A2ASC6:A2ASC6_MOUSE	S D D K W K S T
sp:Q8C570:RAE1L_MOUSE	S D D K W K S T
tr:Q9W2E7:Q9W2E7_DROME	S D D K M K T T
tr:Q7JVX4:Q7JVX4_DROME	S D D K M K T T
sp:Q93454:NPP17_CAEEL	I E D K W N Q M
sp:P40066:GLE2_YEAST	S N D K F Q E M

protein	whole protein ident	simil	domain-based ident	simil	contact-based ident	simil
PDB:3mmy:chainA:P78406	1.0	1.0	1.0	1.0	1.0	1.0
tr:E9PPG9:E9PPG9_HUMAN	0.58	0.59	0.58	0.58	1.0	1.0
sp:P78406:RAE1L_HUMAN	1.0	1.0	1.0	1.0	1.0	1.0
tr:E9PQ57:E9PQ57_HUMAN	0.79	0.82	0.96	0.98	1.0	1.0
sp:Q3SWS8:RAE1L_RAT	0.98	0.99	0.99	1.0	1.0	1.0
tr:A2ASC6:A2ASC6_MOUSE	0.54	0.55	0.54	0.54	1.0	1.0
sp:Q8C570:RAE1L_MOUSE	0.99	1.0	0.99	1.0	1.0	1.0
tr:Q9W2E7:Q9W2E7_DROME	0.65	0.85	0.7	0.9	0.75	0.85
tr:Q7JVX4:Q7JVX4_DROME	0.66	0.87	0.7	0.9	0.75	0.85
sp:Q93454:NPP17_CAEEL	0.5	0.8	0.53	0.81	0.38	0.55
sp:P40066:GLE2_YEAST	0.39	0.76	0.42	0.78	0.38	0.7

Rae1 (FBgn0034646) associated phenotypes

body size defective, lethal - all die before end of second instar larval stage, meiotic cell cycle defective, mitotic cell cycle defective, some die during second instar larval stage

(Information from FlyBase)

rae-1 (WBGene00003803) associated phenotypes

lethal, sterile

(Information from WormBase)

npp-17 (UniProt:Q93454) annotation

Function: Functions as a component of the nuclear pore complex (NPC). NPC components, collectively referred to as nucleoporins (NUPs), can play the role of both NPC structural components and of docking or interaction partners for transiently associated nuclear transport factors. It is specifically important for nuclear mRNA export. (PubMed:12937276).

Subunit: The nuclear pore complex (NPC) constitutes the exclusive means of nucleocytoplasmic transport. NPCs allow the passive diffusion of ions and small molecules and the active, nuclear transport receptor-mediated bidirectional transport of macromolecules such as proteins, RNAs, ribonucleoparticles (RNPs), and ribosomal subunits across the nuclear envelope. Interacts with npp-2. (PubMed:12937276).

Subcellular location: Nucleus, nuclear pore complex (PubMed:12937276).

(Information from UniProt)

Mitogen-activated protein kinase 14

Best gene implication in ageing for this target family came from gene O62618 annotated in UniProt release 2014_02. Annotation GO 8340 (determination of adult lifespan) was Inferred from Mutant Phenotype

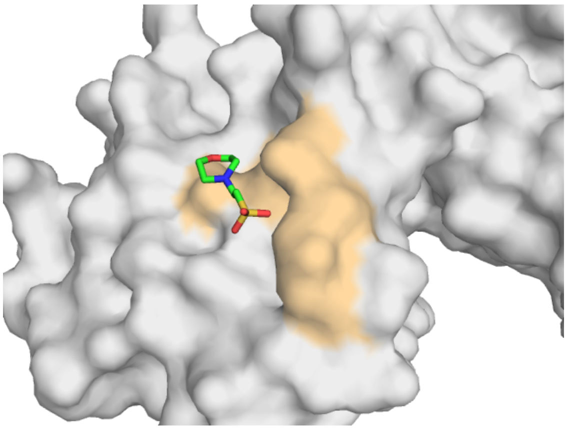

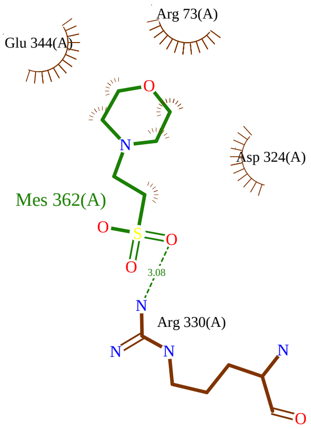

protein	amino acids contacts (binding site)				
PDB:3gcu:chainA:Q16539	R	D	S	R	E
PDB:3huc:chainA:Q16539	R	D	S	R	E
PDB:3hv4:chainA:Q16539	R	D	S	R	E
PDB:3hv5:chainA:Q16539	R	D	S	R	E
tr:L7RSM2:L7RSM2_HUMAN	R	D	S	R	E
sp:Q16539:MK14_HUMAN	R	D	S	R	E
tr:G3V617:G3V617_RAT	R	D	S	R	E
tr:Q56A33:Q56A33_RAT	R	D	S	R	E
sp:P47811:MK14_MOUSE	R	D	S	R	E
tr:Q5U421:Q5U421_MOUSE	R	D	S	R	E
sp:O62618:MK14A_DROME	R	D	S	M	E
tr:E1JIV6:E1JIV6_DROME	R	D	S	M	E
sp:O61443:MK14B_DROME	R	D	S	N	E
tr:E1JHD6:E1JHD6_DROME	K	D	S	R	E
sp:P92208:JNK_DROME	K	D	S	R	E
sp:Q17446:PMK1_CAEEL	R	D	N	R	E
sp:P32485:HOG1_YEAST	K	D	H	A	E

protein	whole protein ident	whole protein simil	domain-based ident	domain-based simil	contact-based ident	contact-based simil
PDB:3gcu:chainA:Q16539	1.0	1.0	1.0	1.0	1.0	1.0
PDB:3huc:chainA:Q16539	1.0	1.0	1.0	1.0	1.0	1.0
PDB:3hv4:chainA:Q16539	1.0	1.0	1.0	1.0	1.0	1.0
PDB:3hv5:chainA:Q16539	1.0	1.0	1.0	1.0	1.0	1.0
tr:L7RSM2:L7RSM2_HUMAN	0.96	0.99	1.0	1.0	1.0	1.0
sp:Q16539:MK14_HUMAN	1.0	1.0	1.0	1.0	1.0	1.0
tr:G3V617:G3V617_RAT	0.99	1.0	0.99	0.99	1.0	1.0
tr:Q56A33:Q56A33_RAT	0.95	0.99	0.99	0.99	1.0	1.0
sp:P47811:MK14_MOUSE	0.99	1.0	0.99	0.99	1.0	1.0
tr:Q5U421:Q5U421_MOUSE	0.96	0.99	0.99	0.99	1.0	1.0
sp:O62618:MK14A_DROME	0.67	0.88	0.63	0.88	0.8	0.79
tr:E1JIV6:E1JIV6_DROME	0.67	0.88	0.63	0.88	0.8	0.79
sp:O61443:MK14B_DROME	0.68	0.89	0.6	0.87	0.8	0.8
tr:E1JHD6:E1JHD6_DROME	0.44	0.78	0.52	0.86	0.8	0.94
sp:P92208:JNK_DROME	0.44	0.78	0.52	0.86	0.8	0.94
sp:Q17446:PMK1_CAEEL	0.61	0.84	0.56	0.83	0.8	0.89
sp:P32485:HOG1_YEAST	0.41	0.68	0.47	0.84	0.4	0.53

Mpk2 (FBgn0015765) associated phenotypes

heat stress response defective, immune response defective, non-enhancer of variegation, non-suppressor of variegation, nutrition conditional, osmotic stress response defective, oxidative stress response defective, partially lethal - majority die, reduced, short lived, starvation stress response defective, stress response defective

(Information from FlyBase)

Mpk2 (UniProt:O62618) annotation

Function: Kinase involved in a signal transduction pathway. May down-regulate insect immunity gene expression after prolonged infection. (PubMed:9417090, PubMed:9584193).
Cofactor:Mg(2+)
Enzyme regulation: Activated by threonine and tyrosine phosphorylation by Mkk3 in response to environmental stress. (PubMed:9417090, PubMed:9584193).
Subcellular location: Nucleus (PubMed:9417090).
Developmental stage: Expressed both maternally and zygotically. Levels are highest at the preblastoderm stage but low levels are present throughout development. (PubMed:9584193).
Domain: The TXY motif contains the threonine and tyrosine residues whose phosphorylation activates the MAP kinases.
Ptm: Dually phosphorylated on Thr-184 and Tyr-186, which activates the enzyme. (PubMed:9417090).

(Information from UniProt)

p38b (FBgn0024846) associated phenotypes
RU486 conditional, circadian rhythm defective, decreased cell size, developmental rate defective, drug conditional, heat stress response defective, immune response defective, locomotor behavior defective, long lived, melanotic mass phenotype, neuroanatomy defective, nutrition conditional, osmotic stress response defective, oxidative stress response defective, partially lethal - majority die, progressive, short lived, small body, starvation stress response defective

(Information from FlyBase)

p38b (UniProt:O61443) annotation
Function: Kinase involved in dpp signal transduction pathway in the process of wing morphogenesis when the levels of dpp are enhanced or inhibited. May down-regulate insect immunity gene expression after prolonged infection. (PubMed:10022918, PubMed:9584193).
Cofactor:Mg(2+)
Enzyme regulation: Activated by threonine and tyrosine phosphorylation by Mkk3. (PubMed:9584193).
Subcellular location: Nucleus.
Tissue specificity: At mid-embryogenesis, highest expression is seen in developing anterior and posterior midguts. Almost ubiquitous expression throughout all development.
Developmental stage: Expressed both maternally and zygotically in the embryo, expression seen in all developmental stages.
Domain: The TXY motif contains the threonine and tyrosine residues whose phosphorylation activates the MAP kinases.
Ptm: Dually phosphorylated on Thr-183 and Tyr-185, which activates the enzyme.

(Information from UniProt)

bsk (FBgn0000229) associated phenotypes
RU486 conditional, cell death defective, chemical resistant, chemical sensitive, conditional, decreased cell growth, dominant, heat sensitive, increased cell death, long lived, neuroanatomy defective, neurophysiology defective, nutrition conditional, oxidative stress response defective, planar polarity defective, progressive, short lived, somatic clone, starvation stress response defective, stress response defective, wound healing defective

(Information from FlyBase)

bsk (UniProt:P92208) annotation
Function: Responds to activation by environmental stress by phosphorylating a number of transcription factors, primarily components of AP-1 such as Jra and also the transcriptional repressor aop, and thus regulates transcriptional activity. Component of the immune response activated by bacterial infection, and is involved in wound healing and in dorsal closure, a morphogenetic movement during embryogenesis. Controls the expression of a phosphatase, puckered, at the edges of wounded epidermal tissue and in the dorsal epithelium during dorsal closure. (PubMed:10433922, PubMed:11784101, PubMed:8946915, PubMed:9224720).
Cofactor:Mg(2+)
Enzyme regulation: Activated by threonine and tyrosine phosphorylation by the dual specificity kinase, hep. Inhibited by dual specificity phosphatase, puckered. (PubMed:10433922).
Subcellular location: Cytoplasm
Tissue specificity: During gastrulation, expression is seen in cells undergoing morphogenetic movements. By stage 9 of embryonic development, expression is ubiquitous. At stages 12-14, expression occurs in epidermis and central nervous system. At stage 15, expression is restricted to ventral nerve cord, brain and some peripheral neurons. In larvae, expression is seen in all imaginal disks, with highest levels in wing and eye disks, and in the CNS. Adults express the protein in fat body and hemocytes. (PubMed:8946915, PubMed:8946916).
Developmental stage: Expressed maternally and zygotically through to adult (male and female). (PubMed:8946915).

Domain: The TXY motif contains the threonine and tyrosine residues whose phosphorylation activates the MAP kinases.
Ptm: Dually phosphorylated on Thr-181 and Tyr-183, which activates the enzyme.

(Information from UniProt)

pmk-1 (WBGene00004055) associated phenotypes

cell stress response variant, drug induced gene expression variant, gene expression level reduced, lethal, life span variant, organism oxidative stress response hypersensitive, pathogen induced gene expression variant, pathogen susceptibility increased, sterile

(Information from WormBase)

pmk-1 (UniProt:Q17446) annotation

Function: Responds to activation by environmental stress and pro- inflammatory cytokines by phosphorylating downstream targets. Functions downstream of the MAPKK sek-1 and the MAPKKK nsy-1 as the MAP kinase required for pathogen resistance. (PubMed:11703092, PubMed:12142542).
Cofactor: Mg(2+) Evidence=(PubMed:11703092);
Enzyme regulation: Activated by phosphorylation on threonine and tyrosine. Inhibited by pyridinyl-imidazole related compounds. (PubMed:11703092).
Domain: The TXY motif contains the threonine and tyrosine residues whose phosphorylation activates the MAP kinases.
Ptm: Dually phosphorylated on Thr-191 and Tyr-193, which activates the enzyme.

(Information from UniProt)

NAD-dependent protein deacetylase sirtuin-2

Best gene implication in ageing for this target family came from gene Q9I7I7 annotated in UniProt release 2014_02. Annotation GO 8340 (determination of adult lifespan) was Inferred from Mutant Phenotype

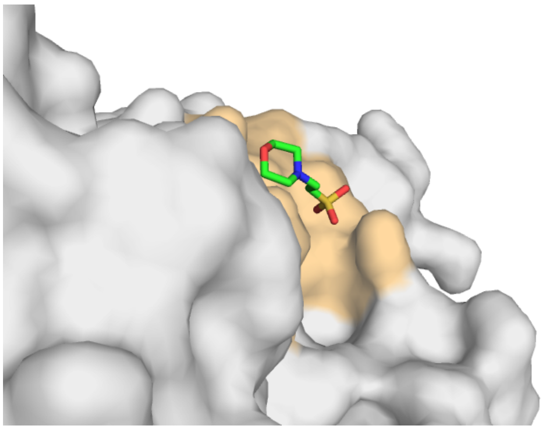

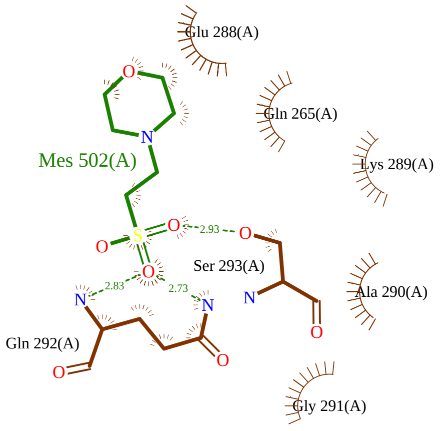

protein	amino acids contacts (binding site)						
PDB:4l3o:chainA:Q8IXJ6	Q	E	K	A	G	Q	S
sp:Q8IXJ6:SIR2_HUMAN	Q	E	K	A	G	Q	S
sp:Q5RJQ4:SIR2_RAT	Q	E	K	T	G	Q	T
tr:Q3UJK6:Q3UJK6_MOUSE	Q	E	K	T	G	Q	T
sp:Q8VDQ8:SIR2_MOUSE	Q	E	K	T	G	Q	T
sp:Q9I7I7:SIRT2_DROME	E	D	A	V	G	Q	A
sp:P53686:HST2_YEAST	A	E	T	V	G	D	-

protein	whole protein ident	simil	domain-based ident	simil	contact-based ident	simil
PDB:4l3o:chainA:Q8IXJ6	1.0	1.0	1.0	1.0	1.0	1.0
sp:Q8IXJ6:SIR2_HUMAN	1.0	1.0	1.0	1.0	1.0	1.0
sp:Q5RJQ4:SIR2_RAT	0.78	0.87	0.88	0.97	0.71	0.82
tr:Q3UJK6:Q3UJK6_MOUSE	0.79	0.87	0.89	0.97	0.71	0.82
sp:Q8VDQ8:SIR2_MOUSE	0.88	0.97	0.9	0.97	0.71	0.82
sp:Q9I7I7:SIRT2_DROME	0.43	0.72	0.47	0.79	0.29	0.58
sp:P53686:HST2_YEAST	0.29	0.57	0.29	0.57	0.29	0.4

Sirt2 (FBgn0038788) associated phenotypes

heat sensitive, lethal - all die before end of pupal stage, some die during pupal stage

(Information from FlyBase)

Sirt2 (UniProt:Q9I7I7) annotation

Function: NAD-dependent protein deacetylase (By similarity). May be involved in the regulation of life span. (, PubMed:17159295).

Cofactor: Zn(2+)Note=Binds 1 zinc ion per subunit. ;

Disruption phenotype: Causes lethality during development. Induced silencing shortens life span. (PubMed:17159295).

(Information from UniProt)
